# Special Issue “Recent Advances in the Synthesis, Functionalization and Applications of Pyrazole-Type Compounds”

**DOI:** 10.3390/molecules26164989

**Published:** 2021-08-18

**Authors:** Vera L. M. Silva, Artur M. S. Silva

**Affiliations:** LAQV-REQUIMTE, Department of Chemistry, University of Aveiro, 3810-193 Aveiro, Portugal

Pyrazoles and their reduced form, pyrazolines, are considered privileged scaffolds in medicinal chemistry, owing to their remarkable biological activities, physicochemical properties and occurrence in many low-molecular-weight compounds present in several marketed drugs (e.g., Celebrex^®^ and Viagra^®^). Pyrazoles are also found in a variety of agrochemicals (fungicides, insecticides, and herbicides) and are versatile compounds for synthetic manipulations. Their structural features (mainly tautomerism, with possible implications in their reactivity), and diverse applications, have stimulated the work of several research groups towards the synthesis and functionalization of pyrazole-type compounds and study of their properties, and has inspired this Special Issue that aims to provide a broad survey of the most recent advances in pyrazole’s chemistry.

In this Special Issue, nine original research articles and seven reviews covering some of the most recent advances in the synthesis, transformation, properties and relevant applications of pyrazoles are reported. Two articles deal with the synthesis and computational study of metallacycles formed by pyrazolate ligands and the coinage metals M = Cu(I), Ag(I) and Au(I). Elguero and Alkorta reported a computational study of metallacycles formed by pyrazolate ligands and the coinage metals (pzM)*_n_* for *n* = 2, 3, 4, 5 and 6 and carried out a comparison with structures reported in the Cambridge Crystallographic Data Center (CCDC). They discussed the most common size of the metallacycles’ ring depending on the metal and analyzed the stability of different ring sizes [1]. By using an unexpected synthetic method, Leznoff and Fusijawa et al. obtained and characterized a silver(I) pyrazolate complex [Ag(µ-L1Clpz)]*_n_* as a coordination polymer, thus broadening the family of silver(I) coordination polymers [2]. El-Faham and Soliman et al. described the synthesis, the molecular structure, using Hirshfeld calculations, and antimicrobial evaluations of three iron(III) complexes obtained by self-assembly of iron(III) chloride and mono- and bis-pyrazolyl-s-triazine ligands. The Fe(III) complexes have shown enhanced antibacterial and antifungal activities as compared to the free ligands [3]. Kayukova et al. described a Boulton–Katritzky rearrangement of 5-substituted phenyl-3-[2-(morpholin-1-yl)ethyl]-1,2,4-oxadiazoles as a synthetic strategy to prepare spiropyrazoline benzoates and chloride. The tested compounds showed moderate to high in vitro antitubercular activity with minimum inhibitory concentration (MIC) values of 1–100 µg/mL. The highest activity in 1 µg/mL and 2 µg/mL on drug sensitive (DS) and multidrug-resistant (MDR) *Mycobacterium tuberculosis* strains, equal to the activity of the basic antitubercular drug rifampicin, was recorded for 2-amino-8-oxa-1,5-diazaspiro[4.5]dec-1-en-5-ium chloride [4]. Potapov et al. described different approaches to the synthesis of dicarboxylic derivatives of bis(pyrazol-1-yl)alkanes which are interesting building blocks for metal-organic frameworks [5]. Svete et al. reported on the synthesis of a series of 12 silica gel-bound enaminones and their Cu(II) complexes, which were used as suitable heterogeneous catalysts in [3+2] cycloadditions of 3-pyrazolidinone-derived azomethine imines to terminal ynones, for the regioselective synthesis of 6,7-dihydro-1*H*,5*H*-pyrazolo[1,2-*a*]pyrazoles [6]. Usami et al. reported their studies on alkylamino coupling reactions at C-4 of 4-halo-1*H*-1-tritylpyrazoles using readily accessible palladium or copper catalysts. They concluded that Pd(dba)_2_-catalyzed reaction of 4-bromo-1*H*-1-tritylpyrazole is suitable for aromatic or bulky amines lacking β-hydrogen atoms, but not for cyclic amines or alkylamines possessing β-hydrogen atoms. On the other hand, the Cu(I)-catalyzed amination using 4-iodo-1*H*-1-tritylpyrazole was found to be favorable for alkylamines possessing β-hydrogen atoms, and not suitable for aromatic amines and bulky amines lacking β-hydrogens, showing the complementarity of the two catalysts [7]. Alam et al. reported the synthesis and antimicrobial studies of 31 novel coumarin-substituted pyrazole derivatives. Some of these compounds have shown potent activity against methicillin-resistant *Staphylococcus aureus* (MRSA) with MIC as low as 3.125 µg/mL and were potent at inhibiting the development of MRSA biofilm and the destruction of preformed biofilm. These are very significant results because MRSA strains have emerged as one of the most alarming pathogens of humans, bypassing human immunodeficiency viruses (HIV) in terms of fatality rate [8]. Wong et al. described the synthesis of novel pyrazolopyridopyridazine diones and *N*-aminopyrazolopyrrolopyridine diones and studied their photoluminescence, solvatofluorism, and quantum yield (Φf). They demonstrated that pyrazolopyridopyridazine diones generally exhibited the stronger fluorescence intensity and possessed a significant substituent effect, particularly for a *m*-chloro substituent, and that a more planar skeletal conformation led to a higher Φf, similar to that of the standard luminol. They also concluded that the introduction of the pyrazole and pyridine chromophores led to an increase in the conjugation and aromaticity of the synthetized compounds when compared with luminol [9]. Current approaches on the synthesis and post-functionalization of pyrazolo[1,5-*a*]pyrimidines were reviewed by Portilla et al., who have also highlighted the anticancer potential and enzyme inhibitory activity of these compounds, thus providing important insights for the rational and efficient design of new drugs bearing the pyrazolo[1,5-*a*]pyrimidine core [10]. Tu et al. described the advances in pyrazole scaffold’s synthesis and functionalization of the last decade (2011–2020) and laid emphasis on the strategies applied in the development of therapeutics for neurodegenerative diseases, particularly for Alzheimer’s disease (AD) and Parkinson’s disease (PD), two of the most serious chronic neurodegenerative diseases. The various pyrazoles described can be regarded as candidate agents for the development of novel neurodegenerative drugs [11]. Brullo et al. highlighted the pharmacological relevance of pyrazolyl-ureas, which are known to possess a wide range of biological activities, such as antipathogenic, antimalarial, anti-inflammatory, antitumoral, among others, and summarized the biological data reported for these compounds from 2000 to date, including patented compounds [12]. Although there are studies on the synthesis of styrylpyrazoles dating back to the 1970s and even earlier, this type of compounds has rarely been studied. Silva et al. focused their attention in this particular type of pyrazoles, containing a styryl (2-arylvinyl) group linked in different positions of the pyrazole backbone, and described their properties, biological activity, methods of synthesis and transformation, highlighting the interest and huge potential for application of this kind of compounds [13]. Lee et al. summarized the recent advances in homocoupling reactions of pyrazoles and other azoles (imidazoles, triazoles and tetrazoles) to highlight the utility of homocoupling reactions in the synthesis of symmetric bi-heteroaryl systems compared with traditional synthesis. Metal-free reactions and transition-metal catalyzed homocoupling reactions were discussed with reaction mechanisms in detail [14]. In the field of energetic materials (EMs), the nitrated pyrazoles have attracted wide attention due to their high heat of formation, high density, tailored thermal stability, and detonation performance, having good applications in explosives, propellants, and pyrotechnics. Zhang and Kou et al. described the recent processes in the synthesis and the physical and explosive performances of the nitrated-pyrazole-based EMs, and analyzed the development trend of these compounds and the concepts for designing prominent high-performance nitropyrazole-based EMs [15]. Kokorekin et al. summarized, for the first time, the poorly studied electrooxidative functionalization of pyrazole derivatives towards the efficient synthesis of C–Cl, C–Br, C–I, C–S and N–N coupling products with applied properties. Important aspects of aromatic hydrogen substitution were discussed, as well as the advantages of electrooxidative functionalization, mainly the predominantly galvanostatic electrolysis mode, the reusability of commercially available electrodes, salts and solvents, and gram-scalability of the processes. The increasing role of cyclic voltammetry as a tool to study mechanisms and to predict the efficiency of the synthesis was highlighted [16].

In conclusion, pyrazoles’ chemistry is undoubtedly a current and pertinent topic of investigation. Several research groups have contributed to the advancement of this topic by designing novel pyrazole-type compounds and developing synthetic strategies for the preparation and post-functionalization of pyrazoles, or by studying their structures, properties and potential applications. Herein, important advances in pyrazoles’ chemistry have been disclosed. We thank all of the authors for their valuable contributions to this Special Issue, all the peer reviewers for their valuable comments, criticisms, and suggestions and the staff members of MDPI for the editorial support.
